# Type 3 Deiodinase and Consumptive Hypothyroidism: A Common Mechanism for a Rare Disease

**DOI:** 10.3389/fendo.2013.00115

**Published:** 2013-09-04

**Authors:** Cristina Luongo, Luigi Trivisano, Fausta Alfano, Domenico Salvatore

**Affiliations:** ^1^Department of Clinical Medicine and Surgery, University of Naples “Federico II,”Naples, Italy

**Keywords:** deiodinase, thyroid hormones, hypothyroidism, thyroid gland, thyroid neoplasms

## Abstract

The major product secreted by the thyroid is thyroxine (T4), whereas most of the biologically active triiodothyronine (T3) derives from the peripheral conversion of T4 into T3. The deiodinase enzymes are involved in activation and inactivation of thyroid hormones (THs). Type 1 and type 2 deiodinase (D1 and D2) convert T4 into T3 whereas D3 degrades T4 and T3 into inactive metabolites and is thus the major physiological TH inactivator. The hypothalamic-pituitary-thyroid axis maintains circulating TH levels constant, while the deiodinases tissue-specifically regulate intracellular thyroid status by controlling TH action in a precise spatio-temporal fashion. Here we review the data related to the recent identification of a paraneoplastic syndrome called “consumptive hypothyroidism,” which exemplifies how deiodinases alter substantially the concentration of TH in blood. This syndrome results from the aberrant uncontrolled expression of D3 that can induce a severe form of hypothyroidism by inactivating T4 and T3 in defined tumor tissue. This rare TH insufficiency generally affects patients in the first years of life, and has distinct features in terms of diagnosis, treatment, and prognosis with respect to other forms of hypothyroidism.

## Introduction

Thyroid hormones (THs) are circulating hormones widely involved in the development and metabolic homeostasis of virtually all mammalian tissues. Alterations in serum TH levels in the fetus and newborns cause significant deficits in experimental animals and in humans (1). The importance of maintaining the TH-dependent transcriptional signature range is supported by the presence of diverse homeostasis checkpoints. In fact, beyond the hypothalamus-pituitary-thyroid axis, which responds to changes in serum levels of thyroid-stimulating hormone (TSH) (2), there are various ways to affect thyroid status in a specific tissue. First, TH exerts its genomic action only in tissues that express a thyroid hormone receptor (TR), and second, it is possible to locally modulate the amount of TH available for cells at a pre-receptoral level. TH transporters and deiodinases are important in determining the availability of TH in a tissue-specific manner.

Although THs are lipophilic molecules, they require specific plasma membrane transporters to enter target cells. Various transporter families have been identified, but only monocarboxylate transporters 8 and 10 (MCT8 and MCT10) and anion transporting polypeptide 1C1 (OATP1C1) have elevated specificity for THs. These proteins are differentially expressed in several tissues: OATP1C1 is expressed in liver, kidney, and brain, MCT8 is highly expressed in liver and brain, and MCT10 is expressed in intestine, kidney, liver, and placenta. A further level of complexity results from the capacity of these transporters to determine the efflux of THs from the intracellular environment to the external milieu (3). The clinical relevance of these transporters is supported by evidence that mutations in the gene encoding MCT8 are associated with elevated serum triiodothyronine (T3) levels and severe sex-linked psychomotor retardation (Allan–Herndon–Dudley syndrome) (4).

Once inside the nucleus, T3 acts through the binding to ligand-dependent transcription factors namely TRs, which bind, mainly as heterodimers with retinoid-X receptors, to TH response elements in target genes (5). TRs are encoded by two genes, THRA and THRB, which are located on chromosomes 17 and 3, respectively. TRα has one T3-binding splice product, TRα1, predominantly expressed in brain, heart, and skeletal muscle, and two non-T3-binding splice products, TRα2 and TRα3, and several additional truncated forms. TRβ has three major T3-binding splice products: TRβ1 is almost ubiquitously expressed; TRβ2 is expressed primarily in the brain, pituitary, retina, and inner ear; and TRβ3 is expressed in kidney, liver, and lung (6).

Selenodeiodinases catalyze the activation (D1 and D2) and inactivation (D3) of the THs thyroxine (T4) and 3,5,3′-triiodothyronine (T3) by removing distinct iodine moieties. D1 and D2 convert thyroxine into the most active metabolic form of TH, T3, by outer ring deiodination; and D3 converts T4 and T3 into the inactive forms rT3 and 3,3′-diiodiothyronine (T2), respectively, by inner ring deiodination. D1 is expressed mostly in the liver, kidney, thyroid, and pituitary; D2 is expressed primarily in the thyroid, central nervous system, pituitary, developing cochlea, brown adipose tissue, and skeletal muscle; D3 is prevalently expressed in many fetal tissues, through the adult it is expressed in placenta, brain, and skin, and to a lesser extent in the pregnant uterus and pancreatic B-cells. The three deiodinases exert different actions: D1 participates in T3 production within the thyroid gland and controls circulating T3 levels, whereas D2 and D3 are active in local deiodination processes. Recent evidence indicates that D1 and D2 are the major sources of circulating T3 in euthyroid humans, with D1 being the major source of circulating T3 in hyperthyroid patients (7). Conversely, D3 inactivates T3 at tissue and plasma level (8). A dramatic example of the potency of the D3 pathways in TH clearance, at both systemic and tissue level, is the newly identified consumptive hypothyroidism syndrome, which is a rare condition resulting from increased D3 production in neoplastic cells and consequent T4 and T3 catabolism.

In this review, we focus on D3 as the cause of consumptive hypothyroidism, a newly recognized clinical entity which often requires close cooperation between clinical endocrinologists, pediatricians, and oncologists.

## Type 3 Deiodinase and Inactivation of Thyroid Hormones

As mentioned, type 3 deiodinase is the physiological inactivator of TH: it catalyzes deiodination on the inner rings of T3 and T4 to produce T2 and rT3, respectively. D3 is a selenoenzyme encoded by the DIO3 gene in humans; it is localized on chromosome 14q32 (9). The DIO3 genomic structure contains a single exon and, at the 3′-UTR, a specific RNA structure, named SECIS (selenocysteine insertion element), that is crucial for the insertion of the selenocysteine residue and for maximal enzymatic catalytic efficiency (10). The DIO3 gene is imprinted, with preferential expression of the paternal allele. It belongs to a cluster of imprinted regions, at the DLK1-DIO3 locus (11).

D3 is an integral membrane protein that exerts its role as a homodimer (12). It is recycled through a system of endosomal clathrin-coated vesicles. This suggests a possible mechanism for D3 reactivation, and furthermore the possibility that this enzyme acts on both extracellular and intracellular pools of T3 and T4 (13). Diverse signals are able to regulate D3 expression *in vitro* and *in vivo*: retinoic acid, serum growth factors, estrogens and progesterone, TGFβ, Wnt-βcatenin, and Shh/Gli2 increase D3 levels, whereas glucocorticoid and growth hormone reduce D3 levels (14, 15).

Recent data from our laboratory implicates p63, a member of the p53 family (16), in D3 regulation (unpublished data). D3 plays the essential role of protecting tissue from excessive TH levels under normal and disease conditions. Indeed, thyrotoxicosis of any cause induces D3, whereas hypothyroidism suppresses D3 (17). D3 activity is elevated during development, a time when circulating fetal TH levels are much lower than those of the mother (18). D3 is widely expressed in such embryonic tissues as liver, cerebral cortex, cardiovascular apparatus, gonads, gut, skin, and urinary tract (19). The human placenta also expresses elevated D3 levels. Within the placenta, D3 blocks the maternal-to-fetal transfer of T4 thereby protecting the fetus from TH excess (20, 21). During late neonatal and adult life, D3 expression is restricted to a few tissues. It has been detected in skin, the central nervous system, and some endocrine glands (22). However, in adult life, D3 expression is reactivated in several disease conditions, namely inflammation, liver regeneration, cardiac hypertrophy and infarct, and cancer (15, 23–25). Several studies have demonstrated that thyroid status affects tumor formation, growth, and metastasis, which suggests that THs are involved in cell transformation. D3 expression has been widely documented in a variety of malignancies; it is turned-on in some malignant cell lines (26) and in a number of human tumors, i.e., oligodendromas, astrocytomas, gliosarcomas, glioblastoma multiforme, TSH-secreting pituitary adenomas, basal cell carcinomas, colon adenomas, and carcinomas (25). It has been postulated that D3 activity is required to facilitate tumor cell proliferation (27).

## Consumptive Hypothyroidism

The paraneoplastic syndrome consumptive hypothyroidism is a rare form of hypothyroidism first identified in newborns with infantile hepatic hemangiomatosis (HHE) (28). The latter is a benign tumor of vascular origin affecting 4–5% of white infants. In most cases, the natural course of HHE is characterized by a rapid proliferation phase during the first 1–2 years of life, and by a slow and steady decline over the next 5–7 years until complete spontaneous involution of the tumor mass. The spectrum of vascular lesions that could be termed “HHE” ranges from benign and self-limiting to aggressive and life-threatening neoplasias. About 10% of cases have aggressive characteristics based on their size, location, and the number of lesions (29). In 2000, Huang et al. reported the first case of severe hypothyroidism (defined as “consumptive hypothyroidism”) in a 6-week-old infant with HHE (28). At the time of diagnosis, TSH level was elevated (156 mUI/ml) and the serum free thyroxine (FT4) concentration was low. Hence, the infant was treated with prednisolone (2 mg/kg) for the hemangioma, and levothyroxine (LT4) replacement (37.5 μg/day) for hypothyroidism, but 16 days later the TSH value was still 256 mUI/ml. At the age of 3 months, the child had intermittent bradycardia and hypothermia, which were probably linked to the hypothyroid status as evidenced by thyroid function tests. In fact, TSH concentration was 177 mUI/ml, serum T4 concentration was 2.5 μg/dl, T3 concentration was low (15 ng/ml), and the level of rT3 was elevated (413 ng/ml). Based on these TH values, replacement hormone therapy was increased to 50 μg/day of LT4, and intravenous administration of 90 μg/day liothyronine was initiated. This therapy resulted in normalization of TSH and T3, but T4 levels remained low. This replacement dose of hormone therapy is very high considering that an athyreotic infant of the same age requires about 7–10 μg/kg/day of LT4 (equivalent to about 3 μg/kg/day of T3), which is about nine times lower than that required by this patient.

After a vertical midline abdominal fasciotomy and embolization of the multiple hemangiomas, the patient died from systemic complications. At that time, the cause of hypothyroidism was not clear, but a markedly elevated serum thyroglobulin concentration argued against a diagnosis of congenital hypothyroidism. Furthermore, biochemical TH parameters suggested that THs were normally synthesized and secreted, but that their rate of degradation was increased. The overexpression of D3 and its activity in the hemangioma tissue were 0.78 pmol of T3 deiodinated/min/mg of protein, a value 7.5 times higher than that normally present in placental tissue, the highest physiological D3-expressing tissue in humans (20).

**Figure 1:**
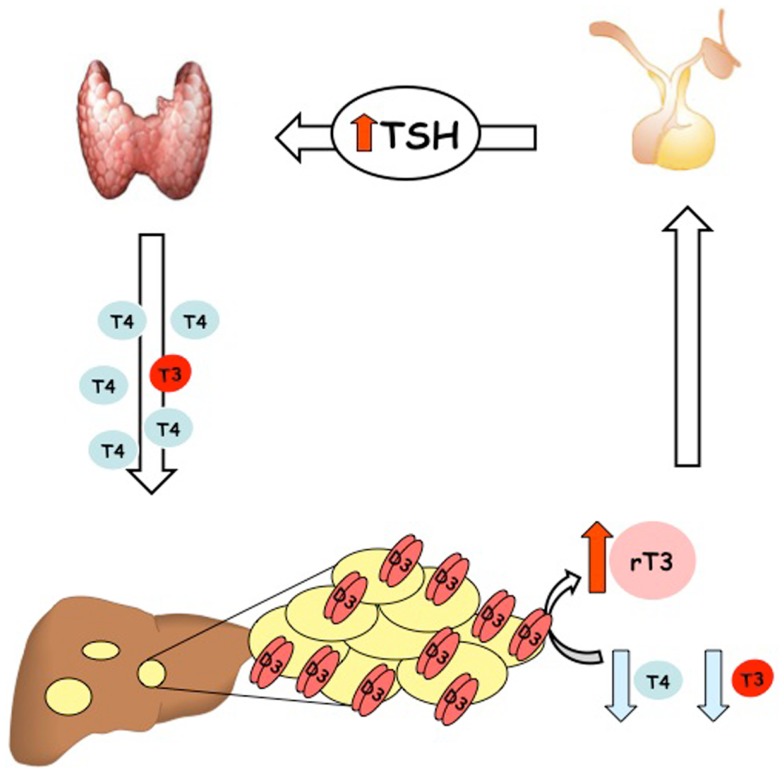
**Schematic illustration of the pathogenesis of condition named “consumptive hypothyroidism**.”

To date, more than 20 cases of consumptive hypothyroidism secondary to neoplasias have been reported (Table 1). An evaluation of all these cases strongly suggests that the development of consumptive hypothyroidism is related to the elevated D3 enzyme, which is in turn directly proportional to the size of the tumor mass and its specific activity, and independent of its localization. Furthermore, most cases described so far support the hypothesis advanced by Huang that D3 activity increases rapidly during the proliferative phase of hemangioma (28). It is also well recognized that consumptive hypothyroidism develops when the catabolic D3 activity exceeds the physiological or exogenous replacement of THs (Figure 1). The direct relationship between consumptive hypothyroidism, tumor size, and D3 activity is demonstrated by the spontaneous remission of hypothyroidism and normalization of serum rT3 concentration upon the natural regression of HHE or after surgical resection of the tumor (Table 1).

**Table 1:** **Summary of published cases of “consumptive hypothyroidism**.”

Patient/age	Sex	Therapy	Outcome	Reference
At birth	F	20 μg/kg/day LT4	Spontaneous regression of hemangioma	Guven et al. (32)
		5 μg/kg/day T3	LT4 replacement therapy for 9 months	
At birth	M	–	Ligation of the hepatic artery	Ayling et al. (35)
			LT4 replacement therapy for 9 months	
3 week	F	25 μg/kg/day LT4	Spontaneous regression of hemangioma	Mouat et al. (33)
			LT4 replacement therapy for 15 months	
4 week	M	25 μg/day LT4	Spontaneous regression of hemangioma	Peters et al. (31)
4 week	M	112 μg/day LT4	Hemangioma recurrence post-pharmacological therapy	Kalpatthi et al. (49)
			Improvement of thyroid function	
6 week	F	15 μg/kg/day LT4	Liver transplantation	Lee et al. (50)
			Euthyroid status post-transplantation	
6 week	M	50 μg/day iv/oral LT4	Embolization of hepatic artery	Huang et al. (28)
		96 μg/day iv T3	Dead	
7 week	M	7 μg/kd/day iv LT4	Embolization of hepaticartery	Mason et al. (36)
		2.5 μg/h iv T3	
7 week	F	7.5 μg/kg/day LT4	Hemangioma recurrence post-pharmacological therapy	Bessho et al. (51)
			Congenital hypothyroidism	
8 week	F	25 μg/day LT4	Dead the day before liver transplantation	Ayling et al. (35)
8 week	F	2 μg/kg/day oral LT4	Hemangioma regression post-pharmacological therapy	Vergine et al. (30)
			LT4 replacement therapy for 10 months	
9 week	M	28 μg/day LT4	Spontaneous regression of hemangioma	Konrad et al. (52)
			LT4 replacement therapy for 3 years	
11 week	M	50 μg/day LT4	Embolization	Jassam et al. (34)
			Dead	
4 months	F	25 μg/day LT4	Ligation of the hepatic artery	Balazs et al. (53)
			LT4 replacement therapy for 2 years	
3 months	F	75 μg/kg/day iv LT4	Liver transplantation	Ayling et al. (35)
		20 μg/kg/day iv	Euthyroid status post-transplantation	
		T3	
4 months	F	T3	Hemangioma regression post-pharmacological therapy	Imteyaz et al. (54)
			LT4 replacement therapy for 10 months	
8 months	M	15 μg/kg/day LT4	Spontaneous regression of skin hemangiomas	Metwalley et al. (46)
10 months	M	120 μg/day LT4	Hemangioma regression post-pharmacological therapy	Cho et al. (37)
			Persistence of hypothyroidism	
21 years	F	88 μg/day oral LT4	Liver transplantation	Huang et al. (38)
			LT4 replacement therapy for 6 weeks	
35 years	F	300 μg/day oral LT4	Partial hepatectomy	Howard et al. (40)
			Improvement of hypothyroidism after thyroidectomy	
54 years	M	1000 μg/day oral LT4	Surgical excision of the tumor malignant fibrous	Huang et al. (38)
			Improvement of hypothyroidism after thyroidectomy	

Recent studies have shed light on the role of D3 in the control of cell proliferation in tumors. In basal cell carcinoma the constitutive activation of the Shh-Gli pathway directly induces D3 mRNA, which in turn reduces TH intracellular activity thereby resulting in increased cyclin D1 expression and keratinocyte proliferation (15). In colon cancer, D3 is a direct target of β-catenin and its activation is a key factor in the regulation of cell proliferation in this tumor (25). Taken together, these findings suggest that local attenuation of TH is an important step in the primary proliferation of some neoplastic cells, which might explain the sustained D3 levels particularly in the initial phase of hemangioma growth.

What induces D3 in hemangioma cells? Data from *in vitro* studies suggest that D3 overexpression in hemangioma is induced by basic fibroblast growth factor and vascular endothelial growth factor. These angiogenic factors play an important role in the pathogenesis of hemangioma. Indeed, recent evidence suggests that propranolol, an antagonist of β2 adrenergic receptors, is able to block tumor growth by inhibiting the expression of these factors in endothelial tumor cells (30).

In most cases, HHE responds to treatment with steroids and/or propanol. If this first-line therapeutic strategy fails, surgery may be necessary, i.e., tumor resection, liver transplantation, or ligation of the hepatic artery (29). The treatment of HHE-associated hypothyroidism can problematic because exogenous hormones are massively converted to inactive forms. The aim of treatment in such cases is to normalize the T4 level, which is critically important particularly for the developing brain during the neonatal period. Hypothyroidism may be very severe, hence large doses of TH are necessary to normalize the T4 level. Doses of LT4 should be increased gradually until TH levels normalize. Since T4 is rapidly converted to the inactive rT3 form, combined therapy with liothyronine may be necessary. Moreover, in severe cases of HHE, parenteral LT4 administration, with or without liothyronine, may be used to bypass the liver and the hemangioma filter (31). Hypothyroidism usually resolves with involution of the tumor, and LT4 treatment may be gradually reduced as involution progresses (32, 33). Therefore, thyroid functions must be frequently monitored to ensure maintenance of the euthyroid status. Monitoring at 6-week intervals is not sufficient for a rapidly developing condition like consumptive hypothyroidism, and weekly monitoring of T4 and T3 levels should be considered particularly in the initial phase of treatment.

Consumptive hypothyroidism is diagnosed based on the detection of D3 activity in the tumor tissue of a patient with biochemical and clinical signs of hypothyroidism. However, HHE biopsy may be a risky procedure due to the high vascularity of the tumor. Hence, consumptive hypothyroidism should be suspected in each HHE patient whose TH values rapidly change especially during the proliferative phase. Usually, the increased D3 activity is mirrored by an elevation of rT3 level associated with a supraphysiological requirement for exogenous hormone and an high serum thyroglobulin level. In such patients, hypothyroidism usually resolves after medical or surgical treatment of HHE.

The differential diagnosis is between congenital or acquired hypothyroidism, TSH-secreting pituitary adenoma and a hormone resistant state. However, a rapid increase of TSH, low levels of T3 and elevated levels of rT3 associated with rapid proliferation of a vascular tumor is typical of consumptive hypothyroidism. Elevated serum thyroglobulin levels, a normal or increased thyroid uptake or the presence of thyroid gland verified by ultrasound can exclude a diagnosis of congenital hypothyroidism (34). Very rarely, a TSH-like factor may be secreted by HHE, as reported in one infant with hypothyroidism and HHE (35). In such cases, the FT4 index is normal in congenital hypothyroidism and elevated in TSH-secreting pituitary adenomas.

In conclusion, given the long-term negative effects of TH alterations on brain and on overall development, thyroid functions must be frequently monitored in patients with HHE. Conversely, since consumptive hypothyroidism has also been described in two infants with asymptomatic HHE (36, 37), HHE should be suspected in all infants with severe unexplained hypothyroidism.

Consumptive hypothyroidism has been described also in adults. The first reported case was a 21-year-old girl with a large hepatic HHE and hypothyroidism manifested with thyroid enlargement, a serum TSH concentration of 26.2 mU/L and normal serum free T4 in the absence of thyroid antibodies (38). Despite the normal FT4 and total T3 levels, serum rT3 was fivefold higher than normal without significant illness or liver failure. The hypothyroidism and goiter resolved after liver transplantation. Subsequently, Ruppe et al. (39) reported the first case of consumptive hypothyroidism associated with a “non-vascular tumor” in an adult who required an elevated dose of LT4 before resection of a large malignant fibrous tumor. Recently, Howard et al. described a 38-year-old athyreotic woman who presented elevated levels of TSH (37.3 mU/L) after the discovery of a large HHE. The patient also had low T3 and elevated rT3 serum levels (40). An interesting aspect of this case was an increase in TSH after partial hepatic resection despite hormone therapy. This may have been due to the hepatic resection itself. Indeed, regenerative processes in hepatic tissue are associated with rapid induction of D3 in post-hepatectomized mice, so one may speculate that hepatic D3 reactivation occurs in humans after liver resection, and this, in turn, causes an increase in TSH levels (24).

## Can Pregnancy be Considered a Mild Form of “Consumptive Hypothyroidism”?

Pregnancy is a physiological condition characterized by changes in thyroidal economy due to a combination of pregnancy-related factors that determine the need for increased TH production. During pregnancy, thyroid function adapts to the increased concentration of T4-binding globulin, the effect of chorionic gonadotropin on maternal thyroid function, the increased requirement of iodine, and the effect of placental deiodinases on TH concentration. Fetal thyroid physiology differs greatly from maternal thyroid physiology, but the two systems interact through the placenta and amniotic fluid. These systems control the transfer of iodine and TH from the mother to the fetus (41). It is important that an appropriate amount of TH be transferred from the mother to the fetus early in gestation because high TH levels are deleterious for developing fetal tissues (42). Therefore, the transplacental supply of TH to the fetus must be finely modulated to ensure appropriate hormone levels. The deiodinases and plasma membrane transporters, which regulate the passage of TH in and out of cells, are major control mechanisms for transplacental TH passage (20).

Fetal thyroid activity begins in mid gestation, at about 18–20 weeks. TSH level peaks at 24–28 weeks, with consequent elevated T4 levels at 35–40 weeks (43). T3 levels remain low during gestation whereas D3 causes an increase in rT3 levels. High D3 expression has been found in human placental syncytiotrophoblasts and cytotrophoblasts, maternal deciduas, and endothelium of fetal vessels (28). During pregnancy, estrogens are potent inducers of D3 (14). Elevation of D3 during pregnancy is necessary to “burn off” the elevated amount of T4 in maternal circulation and acts as a barrier to maternal-fetal exchanges. The injection of 700 μg of T4 into amniotic fluid of pregnant women at term caused an increase of about 13-fold in amniotic fluid T3 concentration, and of about 30-fold in rT3 concentration measured 24 h later (44). Thus, syncytiotrophoblasts, which express high D3 levels, can control the amount and forms of THs taken up by human placenta.

Further evidence that D3 plays a relevant role in pregnancy is the finding that at 6–12 weeks of gestation there is a gradient of T4 from the mother to fetus, which is in the opposite direction of the rT3 gradient. In celomic and amniotic fluid, the concentration of rT3 is 3.8 and 15 times maternal levels, thereby indicating elevated D3 activity (45). The D3 activity in the maternal-fetal unit locally converts most of the T4 derived from the mother into rT3. Low T4 and T3 levels and a high rT3 level is typical of consumptive hypothyroidism. Thus, pregnancy could be considered a physiological form of compensated “consumptive hypothyroidism,” in which the “physiologically increased” expression of D3 increases the inactivation rate of maternal T4 thereby enhancing the need for TH.

## Other Conditions Associated with Elevated D3 Levels

Consumptive hypothyroidism has also been associated with extra-hepatic HHE. In fact, two cases of consumptive hypothyroidism associated with cutaneous hemangioma have been reported in infants (40). Moreover, a case of neonatal hemangioma in the skin, associated with consumptive hypothyroidism, has been reported in an 8-month-old infant (46). The first case of consumptive hypothyroidism secondary to a large parotid hemangioma was reported in 2012 in a child with congenital hypothyroidism and hypoplastic thyroid gland (47). The first case of consumptive hypothyroidism from a non-vascular tumor was reported in 2005 in a patient with a large malignant solitary fibrous tumor, expressing elevated D3 mRNA, and proteins (39). At diagnosis, the patient was administered a supraphysiologic dose of levothyroxine prior to the discovery of the tumor, whose identification suggested that the cause of hypothyroidism could have been the paraneoplastic syndrome.

Notably, D3 is frequently overexpressed in malignant cells, which raises the question “why are circulating TH levels not always perturbed by neoplastic cells?” It is likely that, in most conditions, due to the low D3 expression in tumor cells, and more importantly, to the efficient homeostatic hypothalamic-pituitary-thyroid axis, paraneoplastic D3 is unable to perturb significantly the circulating level of TH.

Finally, tyrosine kinase inhibitors have been recently proposed as therapeutic options for patients with recurrent non-resectable radio-resistant thyroid cancer. In some of these patients undergoing LT4 replacement therapy, treatment with these drugs has been associated with an increase in TSH level. This increase has been associated with the induction (probably at hepatic level) of D3 and, the consequent need to increase the amount of T4 in replacement therapy (48).

## Conclusion

In conclusion, increased catabolism of TH by increased levels of D3 can cause a rare form of acquired hypothyroidism. While this rare syndrome was initially described in association with massive hemangiomas in infants, it was later recognized also in adults, and, in some cases, as a consequence of non-vascular tumors. Although this condition is usually reversible, it requires prompt recognition and immediate TH replacement therapy usually at higher doses than required in other types of hypothyroidism. Furthermore, given that D3 reactivation in tumors is a rather common event in the neoplastic context *in vitro*, and that D3 increase may be a side effect of new pharmacological treatments, it is conceivable that consumptive hypothyroidism may be more frequent than expected. For the clinician, this form of hypothyroidism requires particular attention given its refractory nature toward replacement therapy and the risk of mental and growth retardation in neonatal patients.

## Conflict of Interest Statement

The authors declare that the research was conducted in the absence of any commercial or financial relationships that could be construed as a potential conflict of interest.
